# The efficacy of mycophenolate mofetil in treating Takayasu arteritis: a systematic review and meta-analysis

**DOI:** 10.1007/s00296-017-3704-7

**Published:** 2017-03-31

**Authors:** Danping Dai, YangYang Wang, Haiying Jin, Yiyang Mao, Hao Sun

**Affiliations:** Department of Pharmacy, The Affiliated Hospital of Medicine College, Ningbo University, 247 Renmin Road, Jiangbei District, Ningbo, 315020 Zhejiang China

**Keywords:** Mycophenolate mofetil, Takayasu arteritis, Efficacy, Meta-analysis, Systematic review, Steroid dosage

## Abstract

The purpose of this study is to assess the effectiveness of mycophenolate mofetil (MMF) in treating Takayasu arteritis (TA) patients. Embase, Cochrane Library, Pubmed, Clinicaltrials. Gov and three Chinese literature databases (VIP, CNKI, WanFang) were searched; randomized-controlled trials and observational studies that compared the efficacy before and after treatment with MMF were included. The efficacy outcomes were disease activity, the erythrocyte sedimentation rate (ESR), C-reactive protein (CRP) values and steroid dosage. The results were expressed as mean differences with 95% confidence intervals. Compared with the baseline, there were significant reductions in the ESR (−14.92 [25.35, −4.48]), CRP values (−12.99 [−23.29, −2.68]) and the steroid dosage (−17.64 [−24.89, −10.4]) after the addition of MMF, and the disease tended to stabilize. Therefore, MMF might be an alternative immunosuppressive drug for TA for the control of disease activity and to taper the steroid dosage.

**Electronic supplementary material:**

The online version of this article (doi:10.1007/s00296-017-3704-7) contains supplementary material, which is available to authorized users.

## Introduction

Takayasu arteritis (TA) is a chronic inflammatory disease that mainly affects large arteries such as the aorta and its major branches with an unknown etiopathogenesis [1, 2]. The inflammatory process of Takayasu’s arteritis causes thickening, narrowing or occlusion of the affected vessels and finally results in various symptoms. As well as monetary considerations, patients are concerned about their quality of life. A study demonstrated that the quality of life of TA patients, including both physical and mental components, was worse than that of age-matched, healthy patients [3]. Studies on the management of TA patients are rare, and oral glucocorticoid agents were recommended as first-line therapy [4]. However, many patients require large maintenance steroid doses and are exposed to the risk of chronic toxicity [5]. Nearly, half of all patients will relapse during tapering and, consequently, will require additional immunomodulating drugs, such as azathioprine, cyclophosphamide, and methotrexate [4]. Therefore, we should search for an optimum treatment to improve the therapeutic effect and to reduce disease activity so that the patients’ quality of life is improved.

In recent years, mycophenolate mofetil (MMF) has been widely used as a concomitant drug for TA, and some researchers have studied the application of MMF in TA patients in their countries. MMF may control the disease activity and allow for tapering of the steroid dosage [5, 6]. Nevertheless, to our knowledge, there is no published study assessing the explicit efficacy of treating TA with MMF combined with a steroid systematically. Thus, we performed a systemic review and meta-analysis to evaluate the effectiveness of MMF in patients with TA.

## Methods

We conducted a meta-analysis following the methods specified in the Cochrane Handbook for Systematic Reviews of Intervention [7]. Outcome measures included disease activity (including imaging examinations), the erythrocyte sedimentation rate (ESR), C-reactive protein (CRP) values and the steroid dosage.

### Data sources

Eligible trials were identified through electronic searches (conducted by two independent reviewers, D. D. and Y. W.). Searches were performed in Embase, Cochrane Library, Pubmed, Clinicaltrials. Gov and three Chinese literature databases (VIP, CNKI, WanFang) from their inception until September 2015. The search was limited to English or Chinese articles. The search strategy is shown in Table S1. In addition, the reference lists of eligible studies were also scanned to identify additional relevant studies.

### Study selection

The electronic search results were imported to a management software, and the duplicate results were deleted. Two reviewers (D. D. and Y. W.) independently screened all titles and abstracts for eligible studies. Studies were included if they met the following criteria: (1) TA was diagnosed unequivocally; (2) MMF was used for treatment; (3) the study design was a randomized controlled trial (RCT) or observational study; (4) the study included one of the predefined outcome measures; and (5) the study was published in English or Chinese.

### Data extraction

Two authors extracted data independently (D. D. and Y. W.). Any dispute was settled by discussion or by a third investigator. Study characteristics were extracted from each study, including first author identification, year of publication, sample size, study location, study design, sex, age, duration of TA, duration of MMF therapy, and other concomitant drugs before MMF.

Laboratory parameters (e.g., the ESR value, CRP value, steroid dose, and disease activity) before the first introduction of MMF were extracted as baseline values (before MMF) and were compared to the same parameters extracted at the end of the study (after MMF).

Any drop in the ESR or/and CRP values was considered as efficacy due to the therapy. If patients did not relapse or the disease was stable during the steroid tapering, we also considered the treatment to be effective.

### Quality assessment

The Newcastle–Ottawa Scale (NOS) was adopted to evaluate the quality of the included studies [8].

### Data analysis

The meta-analysis was accomplished by RevMan 5.1 (Cochrane IMS). A fixed effects model was selected and Cochrane Q *χ*² and *I*² statistics were used to estimate the heterogeneity among studies. *I*² values of over 25%, 50% and 75% represent low, moderate and high heterogeneity, respectively [9, 10]. *p* values of 0.05 were used to determine statistical significance. The results were calculated using the Mantel–Haenzsel method and are expressed as mean differences (MD) for continuous outcomes with 95% confidence intervals.

A sensitivity analysis was performed to test the robustness of the main results. We re-analyzed the data using a random effects model. The results for the sensitivity analysis are only reported if the conclusions differed.

## Results

### Literature search

**Fig. 1:**
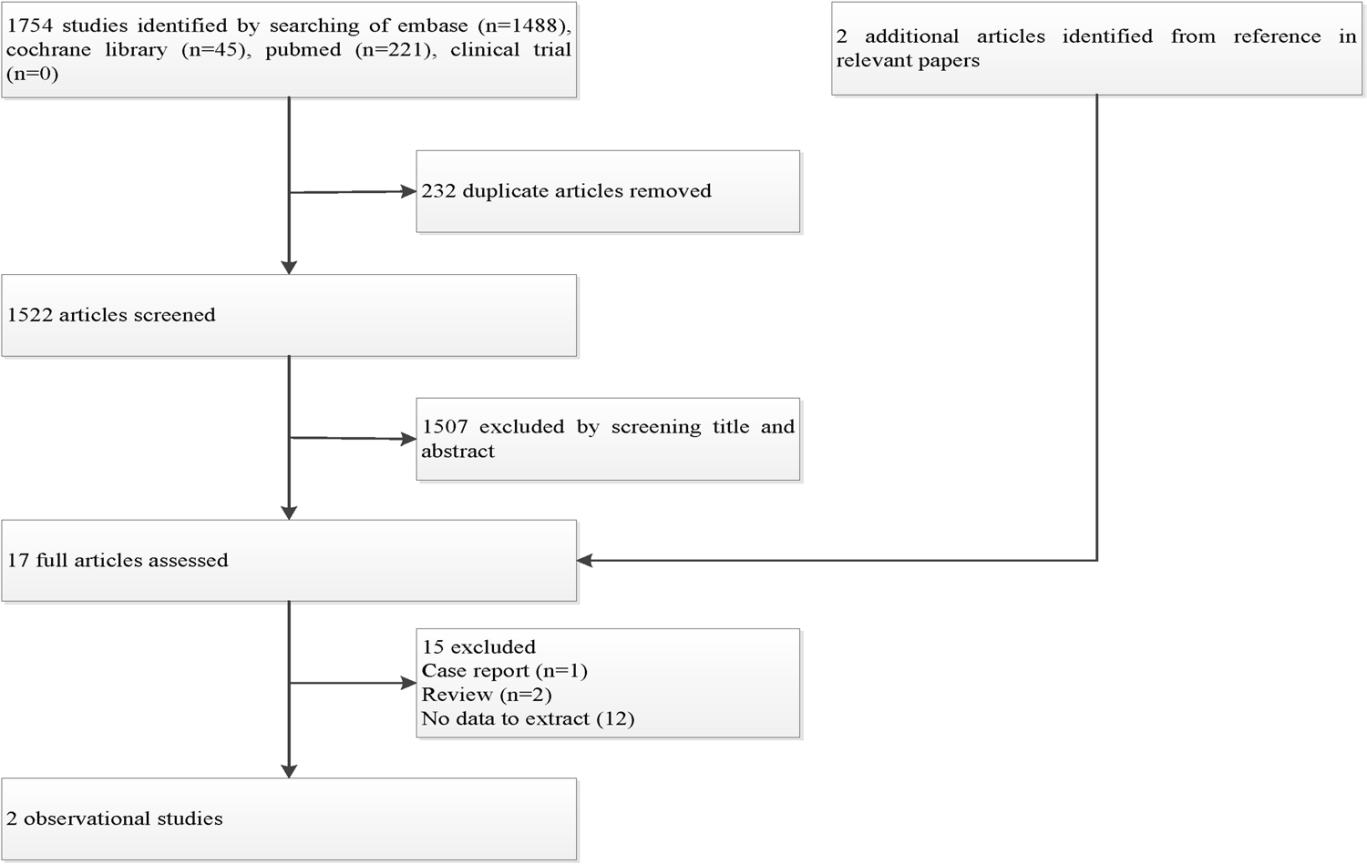
Flow chart of study selection

The study selection process for inclusion is shown in Fig. 1. The electronic searches identified 1524 potentially relevant articles. After initially excluding duplicates and the initial screening, 17 relevant articles were selected, and 15 articles were excluded for the reasons shown in Table S2. A total of 2 articles involving 31 patients were included in the meta-analysis. We did not obtain any additional studies by scanning the reference lists of eligible studies.

### Study characteristics

The characteristics of the included studies are summarized in Table 1. Thirty-one patients diagnosed with TA according to the American College of Rheumatology (ACR) criteria were involved [11]. Of these, two patients abandoned the studies due to their severe adverse events related to MMF [5, 6]. Fifteen patients had received at least one immunosuppressive drug before the administration of MMF (12 azathioprine, 4 methotrexate, and 1 chlorambucil). Because these two studies used different criteria to assess disease activity, we are unable to pool the data.

**Table 1:** Characteristics of the included studies

References	Country, study design	Sample size (male/female)	Age (years)	TA duration (months)	MMF therapy^a^ (months)	Other immunosuppressive drugs before MMF (*n*)
Goel et al. [5]	India, retrospective	21^b^ (2/19)	Mean ± SD: 31.9 ± 13.8	Mean ± SD: 35.5 ± 28.4 Range:1–120	Mean ± SD: 9.6 ± 6.4	Azathioprine (10)
Shinjo et al. [6]	Brazil, prospective	10^c^ (3/7)	Mean ± SD: 29.9 ± 8.9 Range: 18–40	Mean ± SD: 57.5 ± 65.8	Mean ± SD: 23.3 ± 12.1	Methotrexate (4)^d,e^ Azathioprine (2)^d^ Chlorambucil (1)^e^

*TA* Takayasu’s arteritis, *MMF* mycophenolate mofetil

^a^Mycophenolate mofetil as an alternative immunosuppressive drug accompanying steroids in controlling TA disease activity

^b^One patient was not used to evaluate efficacy because of a skin rash, and 11 patients were on steroids alone before MMF

^c^One patient was not used to evaluate efficacy due to a severe headache, and five patients were on steroids alone before MMF

^d^One received methotrexate + azathioprine

^e^One received chlorambucil + methotrexate

### Evaluation of efficacy

**Fig. 2:**
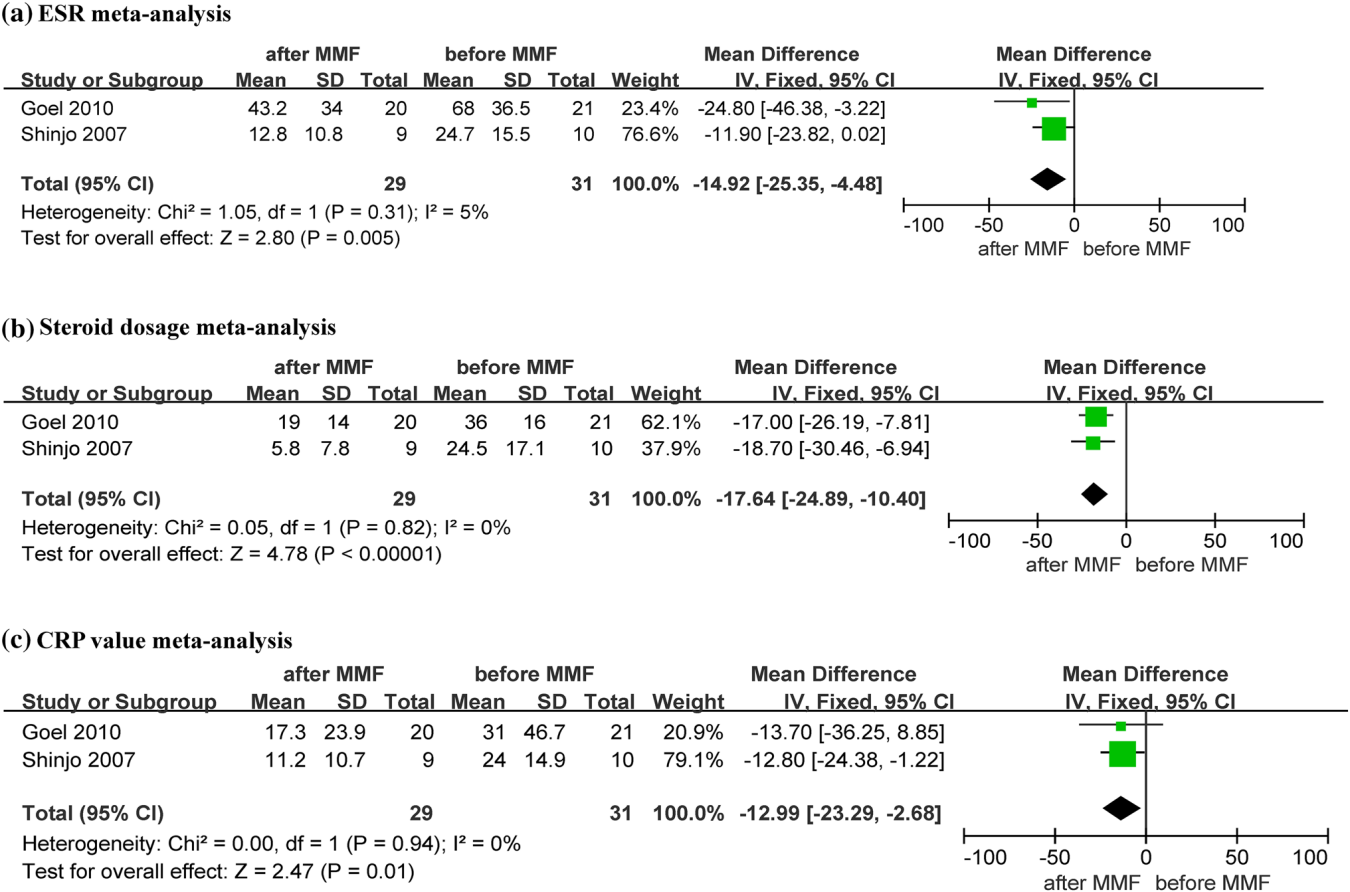
Forest plots

A summary of meta-analysis for efficacy is shown in Table 2; forest plots are shown in Fig. 2. The raw data are shown in Table S3. We performed a fixed effects meta-analysis including 31 patients assigned to the “before” MMF group and 29 patients assigned to the “after” MMF group.

**Table 2:** A summary of the meta-analysis for efficacy

Lab parameter	No. of studies contributing data	MD (95%)	No. of participants of experimental group	No. of participants of control group	*I*² (%)	*p*
ESR	2	−14.92 [25.35, −4.48]	29	31	5	0.005
CRP	2	−12.99 [−23.29, −2.68]	29	31	0	0.01
Steroid dosage	2	−17.64 [−24.89, −10.4]	29	31	0	<0.00001

### Erythrocyte sedimentation rate

Both studies reported a great reduction in the ESR from baseline to the end of the study period, with a mean difference of −14.92 (95% CI −25.35 to −4.48, *p* = 0.005). This result is shown in Fig. 2a. No significant heterogeneity was found in the analysis (*I*² = 5%, *p* = 0.31).

### Steroid dosage

Both studies reported significant decreases in the steroid dosage from baseline to the end of the study. This result is shown in Fig. 2b. The mean difference was −17.64 (95% CI −24.89 to −10.4, *p* < 0.00001). Insignificant heterogeneity was found among the included studies (*I*² = 0%, *P* = 0.82).

### C-reactive protein values

The mean difference of the CRP values was −12.99 (95% CI −23.29 to −2.68, *p* = 0.01). This result is shown in Fig. 2c. There was no significant heterogeneity among the included studies (*I*² = 0%, *p* = 0.94).

### Disease activity

Goel et al. [5] assessed disease activity with Indian Takayasu’s arteritis activity score (ITAS) [12] scoring system, while Shinjo et al. [6] used the National Institutes of Health (NIH) [11] criteria. Regardless of which evaluation standard was used, both studies reported significant improvement in the disease activity, and all patients were stable at the end of the study, except two patients who dropped out of the studies.

### The quality assessment of the included studies

Using the 9-point scoring system, the scores of the included studies were 8 and 9. The results of the specific quality scores from the NOS system are summarized in Table S4.

## Discussion

Primary guidelines recommend immunosuppressive agents, such as azathioprine, cyclophosphamide, and methotrexate as second-line agents [4]. Nevertheless, the long-term administration of such drugs can cause serious side effects. For example, cyclophosphamide can cause cystitis, bladder cancer and infertility (especially in TA patients) [13]. Although less toxic than cyclophosphamide, methotrexate can cause severe bone marrow depression, which may lead to life-threatening infections or spontaneous hemorrhage. These drugs are only to be used carefully when withdrawal from steroids is difficult [14]. Therefore, it is necessary to identify alternative immunosuppressants with lower hepatotoxicity, nephrotoxicity and other severe side effects. Recently, MMF was used to treat TA patients who did not tolerate azathioprine, cyclophosphamide, or methotrexate [1, 2, 5, 6, 15–18], and it achieved a favorable response [2, 5, 6].

Although there are no established specific biological markers to estimate disease, patients with TA often present with higher ESRs and CRP values [19]. Therefore, reduced ESRs and CRP values seem to be a good indicator of disease remission. The results of the present meta-analysis demonstrated that MMF is effective in controlling disease activity and tapering the steroid dosage. Moreover, MMF could significantly decrease the ESR and CRP values. Similar results were reported by Erica Daina [2]. A sensitivity analysis further confirmed that our findings were robust (results shown in Table S5).

However, our study has several limitations. First, although we searched widely, there were only two observational studies included, and the sample size was small (only included 31 patients). Second, because of the lack of data, imaging examination results could not be pooled, although this is an important measurement of long-term efficacy. Third, the use of observational studies in a meta-analysis is liable to the biases and confounding factors that are inherent in the original studies.

In conclusion, MMF might be an alternative immunosuppressive drug for TA incontrolling disease activity and tapering the steroid dosage. However, further research with longer follow-up periods and more participants is needed.

## Electronic supplementary material

Below is the link to the electronic supplementary material.

Supplementary material 1 (DOCX 31 KB)
