# Supplementary material for: The efficacy of mycophenolate mofetil in treating Takayasu arteritis: a systematic review and meta-analysis
Source: Rheumatol Int. 2017 Mar 31;37(7):1083–8. doi: 10.1007/s00296-017-3704-7 (PMC5486790; doi:10.1007/s00296-017-3704-7)
Supplement: Supplementary file 1 — Supplementary material 1 (DOCX 31 KB) [file 296_2017_3704_MOESM1_ESM.docx]

**Supplementary Appendix**

**The efficacy of mycophenolate mofetil in treating Takayasu Arteritis: a systematic review and meta-analysis**

**Danping DAI**

**YangYang WANG**

**Haiying JIN**

**Yiyang Mao**

**Hao Sun**

**Corresponding Author**

**Haiying JIN**

**Address: Department of Pharmacy, The Affiliated Hospital of Medicine College, Ningbo University. 247 Renmin Road, Jiangbei District, Ningbo, Zhejiang, China 315020**

**Tel: 13736053092**

**E-mail: [jinhaiying1975@163.com](mailto:jinhaiying1975@163.com)**

**Contents**

Table S1, Search strategy

Table S2, The reasons for study exclusion

Table S3, The raw outcome measure data before and after treatment with MMF

Table S4, Assessment of quality of studies included in the meta-analysis

Table S5, A summary of the sensitivity analyses using a random model

Table S6, PRISMA checklist

**Table S1: Search strategy**

| **search strategy** | **database** |
| --- | --- |
| (((((((((((((("mycophenolate mofetil ") OR "mycophenolic acid morpholinoethyl ester") OR "RS 61443") OR "RS-61443") OR "Mycophenolate Sodium") OR "Sodium Mycophenolate") OR "Myfortic") OR "Cellcept") OR "mycophenolate mofetil hydrochloride") OR "MMF") OR "MPA") OR "mycophenolic acid") OR "mycophenolic acid 2 morpholinoethyl ester")) AND ((((((((((((((((((((((((("Takayasu Arteritis") OR "Arteritis") OR "TA") OR "Arteritis, Takayasu") OR "Young Female Arteritis") OR "Arteritides, Young Female") OR "Arteritis, Young Female") OR "Female Arteritides, Young") OR "Female Arteritis, Young") OR "Young Female Arteritides") OR "Takayasu Syndrome") OR "Takayasu's Arteritis") OR "Takayasus Arteritis") OR "Arteritis, Takayasu's") OR "Arteritis, Takayasus") OR "Takayasu Disease") OR "Disease, Takayasu") OR "Aortitis Syndrome") OR "Syndrome, Aortitis") OR "Arteritides") OR "Arterial Inflammation") OR "Inflammation, Arterial") OR "Aortitis") OR "Pulseless Disease") OR “aorta arch syndrome”) | pubmed |
| (((((((((((((("mycophenolate mofetil ") OR "mycophenolic acid morpholinoethyl ester") OR "RS 61443") OR "RS-61443") OR "Mycophenolate Sodium") OR "Sodium Mycophenolate") OR "Myfortic") OR "Cellcept") OR "mycophenolate mofetil hydrochloride") OR "MMF") OR "MPA") OR "mycophenolic acid") OR "mycophenolic acid 2 morpholinoethyl ester")) AND ((((((((((((((((((((((((("Takayasu Arteritis") OR "Arteritis") OR "TA") OR "Arteritis, Takayasu") OR "Young Female Arteritis") OR "Arteritides, Young Female") OR "Arteritis, Young Female") OR "Female Arteritides, Young") OR "Female Arteritis, Young") OR "Young Female Arteritides") OR "Takayasu Syndrome") OR "Takayasu's Arteritis") OR "Takayasus Arteritis") OR "Arteritis, Takayasu's") OR "Arteritis, Takayasus") OR "Takayasu Disease") OR "Disease, Takayasu") OR "Aortitis Syndrome") OR "Syndrome, Aortitis") OR "Arteritides") OR "Arterial Inflammation") OR "Inflammation, Arterial") OR "Aortitis") OR "Pulseless Disease") OR “aorta arch syndrome”) | Cochrane |
| **search strategy** | **database** |
| 'mycophenolate mofetil'/exp OR 'mycophenolate mofetil' OR 'mycophenolic acid morpholinoethyl ester' OR 'rs 61443'/exp OR 'rs 61443' OR 'rs-61443'/exp OR 'rs-61443' OR 'mycophenolate sodium'/exp OR 'mycophenolate sodium' OR 'sodium mycophenolate'/exp OR 'sodium mycophenolate' OR 'cellcept'/exp OR 'cellcept' OR 'mycophenolate mofetil hydrochloride'/exp OR 'mycophenolate mofetil hydrochloride' OR 'mmf' OR 'mpa' OR 'myfortic'/exp OR 'myfortic' OR 'mycophenolic acid 2 morpholinoethyl ester'/exp OR 'mycophenolic acid 2 morpholinoethyl ester' OR 'mycophenolic acid'/exp OR 'mycophenolic acid' AND ('takayasu arteritis'/exp OR 'takayasu arteritis' OR 'ta'/exp OR 'ta' OR 'arteritis'/exp OR 'arteritis' OR 'arteritis, takayasu' OR 'pulseless disease'/exp OR 'pulseless disease' OR 'young female arteritis' OR 'arteritides, young female' OR 'arteritis, young female' OR 'female arteritides, young' OR 'female arteritis, young' OR 'young female arteritides' OR 'takayasu syndrome'/exp OR 'takayasu syndrome' OR 'takayasus arteritis' OR 'arteritis, takayasus' OR 'takayasu disease'/exp OR 'takayasu disease' OR 'disease, takayasu' OR 'aortitis syndrome'/exp OR 'aortitis syndrome' OR 'syndrome, aortitis' OR 'arteritides' OR 'arterial inflammation' OR 'inflammation, arterial' OR 'aortitis'/exp OR 'aortitis' OR 'aorta arch syndrome'/exp OR 'aorta arch syndrome') | embase |
| “mycophenolate mofetil” AND “takayasu arteritis” | Clinicaitrial.gov |

**Table S2: The reasons for study exclusion**

| No | Excluded studies | Reason for exclusion |
| --- | --- | --- |
| 1 | Youngstein T, Quinn M, Peters J, Mason JC. Early combination immunosuppression and serial non-invasive imaging improves outcome in takayasu arteritis. Arthritis and RheumatismArthritis Rheum.. 2013. 65: S713-. | No data to extract |
| 2 | Youngstein T, Quinn M, Peters J, Mason JC. Longitudinal analysis suggests clinical outcomes in takayasu arteritis are improved by early combination immunosuppression with serial non-invasive imaging. Annals of the Rheumatic DiseasesAnn. Rheum. Dis.. 2014. 73: -. | No data to extract |
| 3 | Youngstein T, Mason J. Takayasu arteritis-An outcome study in a UK cohort. Presse MedicalePresse Med.. 2013. 42(4): 677- | No data to extract |
| 4 | Youngstein T, Mehta P, Mason J. Takayasu arteritis: A ten year longitudinal study in the UK. RheumatologyRheumatology (UK). 2011. 50: iii146-.[1, 2] | MMF was not used and no data to extract |
| 5 | Goel R, Danda D, Kumar S, Joseph G. A single centre experience of 40 children with Takayasu arteritis from India. Presse MedicalePresse Med.. 2013. 42(4): 728-729. | No data to extract |
| 6 | Freitas DS, Camargo CZ, Mariz HA, Arraes AED, De Souza A.W.S. Takayasu arteritis: Assessment of response to medical therapy based on clinical activity criteria and imaging techniques. Rheumatology InternationalRheumatol. Int.. 2012. 32(3): 703-709. | No data to extract |
| 7 | Liang P, Hoffman GS. Advances in the medical and surgical treatment of Takayasu arteritis. Current Opinion in RheumatologyCurr. Opin. Rheumatol.. 2005. 17(1): 16-24. | No data to extract |
| 8 | Schmidt J, Kermani TA, Bacani AK, et al. Diagnostic features, treatment, and outcomes of Takayasu arteritis in a US cohort of 126 patients. Mayo Clinic ProceedingsMayo Clin. Proc.. 2013. 88(8): 822-830. | No data to extract |
| 9 | Keser G, Direskeneli H, Aksu K. Management of Takayasu arteritis: A systematic review. Rheumatology (United Kingdom)Rheumatology. 2014. 53(5): 793-801. | This is a review and No data to extract |
| No | Excluded studies | Reason for exclusion |
| 10 | Maksimowicz-McKinnon K, Clark TM, Hoffman GS. Limitations of therapy and a guarded prognosis in an American cohort of Takayasu arteritis patients. Arthritis and RheumatismArthritis Rheum.. 2007. 56(3): 1000-1009. | No data to extract |
| 11 | Cong X-L, Dai S-M, Feng X, et al. Takayasu's arteritis: Clinical features and outcomes of 125 patients in China. Clinical RheumatologyClin. Rheumatol.. 2010. 29(9): 973-981. | No data to extract |
| 12 | Mustafa KN, Hadidy A, Sweiss NJ. Clinical and radiological features of Takayasu's arteritis patients in Jordan. Rheumatology InternationalRheumatol. Int.. 2010. 30(11): 1449-1453. | No data to extract |
| 13 | Daina E, Schieppati A, Remuzzi G. Mycophenolate mofetil for the treatment of takayasu arteritis: Report of three cases. Annals of Internal MedicineAnn. Intern. Med.. 1999. 130(5): 422-426. | This is a case report |
| 14 | Ito I. Medical treatment of Takayasu arteritis. Heart Vessels. 7(1): 133-137. | This is a review |
| 15 | Hu W, Liu C, Xie H, Chen H, Liu Z, Li L. Mycophenolate mofetil versus cyclophosphamide for inducing remission of ANCA vasculitis with moderate renal involvement. Nephrol Dial Transplant. 2008. 23(4): 1307-12. | Do not accord with the disease which is predefined |

**Table S3: The raw outcome measure data before and after treatment with MMF**

| **Reference** | **Disease activity** | | | **Steroid dosage**  **(mg/day)** | | **ESR**  **(mm/h)** | | **CRP**  **(mg/l)** | |
| --- | --- | --- | --- | --- | --- | --- | --- | --- | --- |
|  | **criteria** | **Before MMF** | **After MMF** | **Before MMF** | **After MMF** | **Before MMF** | **After MMF** | **Before MMF** | **After MMF** |
| Goel  2010 | ITAS | Median:7  Range:0-19 | Median:1  Range:0-7 | Mean±SD:  36 ±16 | Mean±SD: 19±14 | Mean±SD: 68±36.5 | Mean±SD: 43.2±34 | Mean±SD: 31±46.7 | Mean±SD: 17.3±23.9 |
| Shinjo  2007 | NIH | 10^f^ | 1^f^ | Mean±SD: 24.5±17.1 | Mean±SD: 5.8±7.8 | Mean±SD: 24.7±15.5 | Mean±SD: 12.8±10.8 | Mean±SD: 24.0±14.9 | Mean±SD: 11.2±10.7 |

ESR: erythrocyte sedimentation rate

CRP: C-reactive protein

An ITAS value of 0 was taken as the complete absence of disease activity.

f: The number of patients who had active disease.

**Table S4. Assessment of quality of studies included in the meta-analysis**

| **Cohort studies** | **Selection** | | | | **Comparability** | | **Outcome** | | | **Total sore** |
| --- | --- | --- | --- | --- | --- | --- | --- | --- | --- | --- |
|  | **1** | **2** | **3** | **4** | **5A** | **5B** | **6** | **7** | **8** |  |
| Goel 2010 | * | * | * | * | * | * | * | _ | * | 8 |
| Shinjo 2007 | * | * | * | * | * | * | * | * | * | 9 |

Selection: 1 = representativeness of the exposed cohort; 2 = selection of the non-exposed cohort; 3 = ascertainment of exposure;

4 = demonstration that the outcome of interest was not present at the start of the study

Comparability: 5A = study controls for diagnostic criteria; 5B = study controls for any additional factor

Outcome: 6 = assessment of outcome; 7= was follow-up long enough for outcomes to occur (follow-up time > 1 year was assigned a score of 1);

**Table S5: A summary of the sensitivity analyses using a random model**

| Lab parameter | No. of studies  contributing data | MD (95%) | No. of participants of  experimental group | No. of participants of  control group | I²(%) | P |
| --- | --- | --- | --- | --- | --- | --- |
| ESR | 2 | -15.08[-25.99, -4.18] | 29 | 31 | 5 | 0.007 |
| CRP | 2 | -12.99[-23.29, -2.68] | 29 | 31 | 0 | 0.01 |
| Steroid dosage | 2 | -17.64[-24.89, -10.4] | 29 | 31 | 0 | <0.00001 |

**Table S6: PRISMA checklist**

| **Section/topic** | **#** | | **Checklist item** | | **Reported on page #** | |  |
| --- | --- | --- | --- | --- | --- | --- | --- |
| **TITLE** | | | | |  | |  |
| Title | 1 | | The efficacy of mycophenolate mofetil in treating Takayasu Arteritis: a systematic review and meta-analysis | | title page | |  |
| **ABSTRACT** | | | | |  | |  |
| Structured summary | 2 | | Background, objectives, data sources, participants, and interventions; study appraisal and synthesis methods, results, conclusions and implications of key findings. | | title page | |  |
| **INTRODUCTION** | | | | |  | |  |
| Rationale | 3 | | We conducted a meta-analysis following the methods specified in the Cochrane Handbook for Systematic Reviews of Intervention. | | 2 | |  |
| Objectives | 4 | | Participants: Takayasu Arteritis patients  Interventions: mycophenolate mofetil + steroid  Comparisons: other immunosuppressive drugs + steroid or steroid  Outcomes: disease activity (including imaging examinations), the erythrocyte sedimentation rate (ESR), C-reactive protein (CRP) values and the steroid dosage  Study design: observational studies or randomized controlled trial | | 2 | |  |
| **METHODS** | | | | |  | |  |
| Protocol and registration | 5 | | Our study didn’t have been registered. | |  | |  |
| Eligibility criteria | 6 | | (1) TA was diagnosed unequivocally; (2) MMF was used for treatment; (3) the study design was a randomized controlled trial (RCT) or observational study; (4) the study included one of predefined outcome measures; and (5) the study was published in English or Chinese. | | 4-5 | |  |
| Information sources | 7 | | Searches were performed in Embase, Cochrane Library, Pubmed, Clinicaltrials. Gov and 3 Chinese literature databases (VIP, CNKI, WanFang) from their inception until September 2015. In addition, the reference lists of eligible studies were also scanned to identify additional relevant studies. | | 4 | |  |
| Search | 8 | | (((((((((((((("mycophenolate mofetil ") OR "mycophenolic acid morpholinoethyl ester") OR "RS 61443") OR "RS-61443") OR "Mycophenolate Sodium") OR "Sodium Mycophenolate") OR "Myfortic") OR "Cellcept") OR "mycophenolate mofetil hydrochloride") OR "MMF") OR "MPA") OR "mycophenolic acid") OR "mycophenolic acid 2 morpholinoethyl ester")) AND ((((((((((((((((((((((((("Takayasu Arteritis") OR "Arteritis") OR "TA") OR "Arteritis, Takayasu") OR "Young Female Arteritis") OR "Arteritides, Young Female") OR "Arteritis, Young Female") OR "Female Arteritides, Young") OR "Female Arteritis, Young") OR "Young Female Arteritides") OR "Takayasu Syndrome") OR "Takayasu's Arteritis") OR "Takayasus Arteritis") OR "Arteritis, Takayasu's") OR "Arteritis, Takayasus") OR "Takayasu Disease") OR "Disease, Takayasu") OR "Aortitis Syndrome") OR "Syndrome, Aortitis") OR "Arteritides") OR "Arterial Inflammation") OR "Inflammation, Arterial") OR "Aortitis") OR "Pulseless Disease") OR “aorta arch syndrome”) | | Table S1 | |  |
| Study selection | 9 | | The electronic search results were imported to a management software, and the duplicate results were deleted. Two reviewers (D. D. and Y. W.) independently screened all titles and abstracts for eligible studies. Studies were included if they met the eligiblility criteria. | | 4-5 | |  |
| Data collection process | 10 | | Two authors extracted data independently (D. D. and Y. W.). Any dispute was settled by discussion or by a third investigator. | | 5 | |  |
| Data items | 11 | | the ESR, CRP value, steroid dose | | 4 | |  |
| Risk of bias in individual studies | 12 | | The Newcastle-Ottawa Scale (NOS) | | 5 | |  |
| Summary measures | 13 | | The results are expressed as mean differences (MD) for continuous outcomes with 95% confidence intervals | | 6 | |  |
| Synthesis of results | 14 | | The meta-analysis was accomplished by RevMan 5.1 (Cochrane IMS). A fixed effects model was selected and Cochrane Qχ² and I² statistics were used to estimate the heterogeneity among studies. I² values of over 25%, 50% and 75% represent low, moderate and high heterogeneity, respectively. P values of 0.05 were used to determine statistical significance. | | 6 | |  |
|  |  | |  | |  | |  |
| **Section/topic** | | **#** | | **Checklist item** | | **Reported on page #** | |
| Risk of bias across studies | | 15 | | Risk of bias across studies have not been accessed because our study only included 2 articles. | |  | |
| Additional analyses | | 16 | | sensitivity analysis | | Table S5 | |
| **RESULTS** | | | | | |  | |
| Study selection | | 17 | | The study selection process for inclusion is shown in Fig 1. The electronic searches identified 1524 potentially relevant articles. After initially excluding duplicates and the initial screening, 17 relevant articles were selected, and 15 articles were excluded for the reasons shown in Table 2. A total of 2 articles involving 31 patients were included in the meta-analysis. We did not obtain any additional studies by scanning the reference lists of eligible studies. | | 6 | |
| Study characteristics | | 18 | | Table 1 | |  | |
| Risk of bias within studies | | 19 | | Table S5 | |  | |
| Results of individual studies | | 20 | | Fig 2 | |  | |
| Synthesis of results | | 21 | | Table 2 | |  | |
| Risk of bias across studies | | 22 | | Risk of bias across studies have not been accessed because our study only included 2 articles. | |  | |
| Additional analysis | | 23 | | Table S4 | | 14 | |
| **DISCUSSION** | | | | | |  | |
| Summary of evidence | | 24 | | According to the GRADE Guideline, the strength of evidence for each main outcome was high. The results might provide an important evidence for clinical practice | |  | |
| Limitations | | 25 | | First, although we searched widely, there were only two observational studies included, and the sample size was small (only included 31 patients). Second, because of the lack of data, imaging examination results couldn’t be pooled, although this is an important measurement of long-term efficacy. Third, the use of observational studies in a meta-analysis is liable to the biases and confounding factors that are inherent in the original studies. | | 9 | |
| Conclusions | | 26 | | MMF might be an alternative immunosuppressive drug for TA incontrolling disease activity and tapering the steroid dosage. | | 9 | |
| **FUNDING** | | | | | |  | |
| Funding | | 27 | | This study was not supported by any funding. | |  | |
